# Cardiovascular magnetic resonance imaging of functional and microstructural changes of the heart in a longitudinal pig model of acute to chronic myocardial infarction

**DOI:** 10.1186/s12968-021-00794-5

**Published:** 2021-09-20

**Authors:** Christian T. Stoeck, Constantin von Deuster, Maximilian Fuetterer, Malgorzata Polacin, Conny F. Waschkies, Robbert J. H. van Gorkum, Mareike Kron, Thea Fleischmann, Nikola Cesarovic, Miriam Weisskopf, Sebastian Kozerke

**Affiliations:** 1 Institute for Biomedical Engineering, University and ETH Zurich, Gloriastrasse 35, 8092 Zurich, Switzerland; 2 Institute of Diagnostic and Interventional Radiology, University Hospital Zurich, Zurich, Switzerland; 3 Division of Surgical Research, University Hospital Zurich, Zurich, Switzerland; 4 Institute of Translational Cardiovascular Technologies, ETH Zurich, Zurich, Switzerland

**Keywords:** Chronic myocardial infarction, Microstructural CMR, Functional CMR, CMR Relaxometry, T_2_ mapping, T_1_ mapping, ECV, DCE, LGE, Cardiac DTI

## Abstract

**Background:**

We examined the dynamic response of the myocardium to infarction in a longitudinal porcine study using relaxometry, functional as well as diffusion cardiovascular magnetic resonance (CMR). We sought to compare non contrast CMR methods like relaxometry and in-vivo diffusion to contrast enhanced imaging and investigate the link of microstructural and functional changes in the acute and chronically infarcted heart.

**Methods:**

CMR was performed on five myocardial infarction pigs and four healthy controls. In the infarction group, measurements were obtained 2 weeks before 90 min occlusion of the left circumflex artery, 6 days after ischemia and at 5 as well as 9 weeks as chronic follow-up. The timing of measurements was replicated in the control cohort. Imaging consisted of functional cine imaging, 3D tagging, T2 mapping, native as well as gadolinium enhanced T1 mapping, cardiac diffusion tensor imaging, and late gadolinium enhancement imaging.

**Results:**

Native T1, extracellular volume (ECV) and mean diffusivity (MD) were significantly elevated in the infarcted region while fractional anisotropy (FA) was significantly reduced. During the transition from acute to chronic stages, native T1 presented minor changes (< 3%). ECV as well as MD increased from acute to the chronic stages compared to baseline: ECV: 125 ± 24% (day 6) 157 ± 24% (week 5) 146 ± 60% (week 9), MD: 17 ± 7% (day 6) 33 ± 14% (week 5) 29 ± 15% (week 9) and FA was further reduced: − 31 ± 10% (day 6) − 38 ± 8% (week 5) − 36 ± 14% (week 9). T2 as marker for myocardial edema was significantly increased in the ischemic area only during the acute stage (83 ± 3 ms infarction vs. 58 ± 2 ms control p < 0.001 and 61 ± 2 ms in the remote area p < 0.001). The analysis of functional imaging revealed reduced left ventricular ejection fraction, global longitudinal strain and torsion in the infarct group. At the same time the transmural helix angle (HA) gradient was steeper in the chronic follow-up and a correlation between longitudinal strain and transmural HA gradient was detected (r = 0.59 with p < 0.05). Comparing non-gadolinium enhanced data T2 mapping showed the largest relative change between infarct and remote during the acute stage (+ 33 ± 4% day 6, with p = 0.013 T2 vs. MD, p = 0.009 T2 vs. FA and p = 0.01 T2 vs. T1) while FA exhibited the largest relative change between infarct and remote during the chronic follow-up (+ 31 ± 2% week 5, with p = N.S. FA vs. MD, p = 0.03 FA vs. T2 and p = 0.003 FA vs. T1). Overall, diffusion parameters provided a higher contrast (> 23% for MD and > 27% for FA) during follow-up compared to relaxometry (T1 17–18%/T2 10–20%).

**Conclusion:**

During chronic follow-up after myocardial infarction, cardiac diffusion tensor imaging provides a higher sensitivity for mapping microstructural alterations when compared to non-contrast enhanced relaxometry with the added benefit of providing directional tensor information to assess remodelling of myocyte aggregate orientations, which cannot be otherwise assessed.

**Supplementary Information:**

The online version contains supplementary material available at 10.1186/s12968-021-00794-5.

## Background

The loss of functional cardiac tissue due to myocardial infarction (MI) can lead to ventricular remodeling including myofiber disarray, myocardial wall thinning and dilatation compromising left ventricular (LV) function [1, 2]. Without timely treatment, these conditions often progress towards clinical heart failure [3, 4].

The response to an ischemic event has been characterized as a dynamic process [5]. During the first hours, reperfusion-related edema leads to swelling of injured myocardium, followed by a period of reduced swelling during the subsequent days with collagen deposition, leading to a second wave of inflammatory edema associated with healing [6]. The degradation of cell membranes and vasculature in the ischemic region results in an elevated extracellular volume fraction (ECV) [7], which increases the residence time of gadolinium-based contrast agents as detected by late gadolinium-enhancemed (LGE) imaging [8]. The successive LV remodelling is characterized by ventricle enlargement and increase in LV sphericity [9].

Non-viable infarcted tissue leads to an increase in the cardiac workload of the remaining viable myocardium and subsequently to compensatory hypertrophy [10]. In addition, ventricular dilation may be considered as a compensatory response to maintain stroke volume in the presence of declining ejection fraction through the Frank–Starling mechanism [11]. While this compensatory process may be viewed as a positive response to keep up blood supply to the systemic circulation, it leads to increasing systolic and diastolic wall stresses, which stimulate further ventricular remodelling [12]. Among patients studied 2 to 4 weeks after an acute MI, those who developed a spherical ventricle appeared to have a greater propensity for the development of heart failure [13]. Similarly, an increase of LV volume is associated with poor long-term prognosis [14].

With changes in LV shape, alterations in function have been reported: Global longitudinal strain (GLS), global radial strain (GRS) and global circumferential strain (GCS) were found to be significantly decreased in magnitude in patients with MI when compared with controls [15]. While all three strain indices are impaired in MI patients, GLS was found to be the strongest predictor for major adverse cardiac events (MACE) [16] and most consistently detected the change in remodelled LVs during follow-up across different studies [17]. Besides characterizing the tissue deformation by strain measurements, rotation, twist and torsion have been analysed as functional marker in MI patients. Patients who developed LV remodeling at a 6 month follow-up showed significantly impaired peak subepicardial and subendocardial twist compared to patients without remodeling [18]. The impact on LV rotational patterns is, however, dependent on infarct location. The apical rotation was found to be lower in patients with acute anterior compared with acute inferior MI, while vice versa for basal rotation. As consequence, overall LV torsion was not significantly different between the two infarct groups [19].

The changes in shape and function coincide with changes in tissue composition that can be assessed by cardiovascular magnetic resonance (CMR) relaxometry. Edema present during the acute stage after myocardial ischemia/reperfusion injury [6] is associated with significantly increased T2 relaxation times. During the chronic post-MI stages, dead cardiomyocytes are replaced by extracellular matrix resulting in chronically increased ECV. By measuring pre- and post-contrast T1 values in conjunction with sampling the patient’s haematocrit, this increase in ECV can be quantified [20].

Complementary to measuring changes in relaxation times, cardiac diffusion tensor imaging (cDTI) has become an important tool to non-destructively probe changes of microstructure in the anisotropic myocardium. cDTI provides parameters such as mean diffusivity (MD) and fractional anisotropy (FA), thereby granting insights into the structural integrity of tissue, as well as spatially directional parameters such as the inclination angle of cardiomyocyte aggregates, also known as helix angle. In the realm of MI, an elevated MD and a reduced FA was found within the affected region due to loss of tissue integrity resulting in fewer restrictions to water molecules and providing a prolonged mean free pathway [21–23]. In the chronic stage, structural remodelling in the remote zone was found in pigs with permanent left-anterior descending (LAD) or left circumflex (LCX) coronary artery occlusion, reflected in an increase of left-handed myocyte aggregates (epicardium) in conjunction with a decrease in right-handed myocyte aggregates (endocardium) [22]. However, only differences in the cases of LAD occlusion were statistically significant. Similar studies in sheep [24] and patients [25] with chronic MI did not find statistically significant changes in the handedness of the microstructural architecture. Furthermore, the myocyte propagation angle has shown good spatial correlation with LGE imaging in mice and patients with MI. Areas with scar showed increased variability when tracking the principal direction of myocyte aggregates [26].

Relaxometry as well as cDTI both assess tissue alterations that occur at the microscopic level. While T2 and T1 mapping reflect changes in spin–spin and spin–lattice relaxation that are influenced by water content and macromolecular environment, while cDTI is sensitive to changes of water self-diffusion that is governed by the presence and architecture of restrictions such as cell membranes.

In the present work, we present a longitudinal swine study to compare relaxometry and cDTI during the dynamic response of the myocardium to MI from the healthy to the acute and chronic MI stages. Using the longitudinal multi-parametric study design, we furthermore exploit the capability of cDTI to derive information on myocyte aggregate orientation to potentially link microstructural changes due to microstructural remodelling with functional alterations in the infarcted heart over time.

## Methods

Nine healthy female swine (Swiss Large White “Edelschwein”, 30–35 kg) were imaged on a clinical 1.5T CMR system (Achieva, Philips Healthcare, Best, The Netherlands) equipped with a gradient system delivering 80 mT/m per physical axis at a slew rate of 100 T/m/s.

The study was approved by the veterinary office of the canton of Zurich under licence: ZH072/16.

### Animal handling

All experimental procedures were performed under general anaesthesia. The pigs were pre-medicated with an intramuscular injection of ketamine (15 mg/kg, Ketasol^®^-100, Graeub AG, Bern Switzerland), azaperone (2 mg/kg, Stresnil^®^, Elanco Tiergesundheit AG, Basel Switzerland) and atropine (0.05 mg/kg, Atropin 1% Kantonsapotheke, Zurich Switzerland). An intravenous catheter was placed and anaesthesia was deepened with propofol (Propofol-Lipuro 2 mg/kg, B. Braun Medical AG, Sempach, Switzerland) followed by orotracheal intubation. Percutaneous sheaths (5F) were introduced into the femoral arteries and veins. 100 IU/kg body weight (BW) heparin was administered intravenously and was repeated every hour. General anaesthesia was maintained with isoflurane (2–3%) by positive pressure ventilation with 100% oxygen. Heart rate and rhythm and variability (ECG), inspiratory and expiratory gases (CO_2_, O_2_, isoflurane), pulse oximetry, temperature, direct arterial blood pressure and urine output were monitored continuously throughout the procedure. Arterial blood gas analysis was performed hourly to assess gases (pO_2_, pCO_2_), electrolyte balance (Na^+^, K^+^, Ca^2+^, Cl^−^), haematocrit as well as glucose, lactate and creatinine. Additionally, high-sensitive Troponin T (hsTnT) levels were measured according to study time points. Analgesia was provided by administration of buprenorphine (0.01 mg/kg) every 4–6 h throughout the procedure and by meloxicam (0.4 mg/kg) orally for 2 days.

Before MI induction, animals received antiarrhythmic medication [150 mg amiodarone (Cordarone^®^, Sanofi-Aventis, Paris, France) dissolved in 100 ml 5% glucose solution]. For cardiac synchronization of the imaging sequences, ECG electrodes (Quatrode patch, In-Vivo Corporation, Gainesville, Florida, USA) were placed onto the chest between the front limbs.

After the final CMR measurement (week 9) the animals were euthanized by an overdose of pentobarbital (75 mg/kg BW iv) in deep anaesthesia.

### Study protocol

Animals were grouped into MI (N = 5) and control (N = 4) cohorts. Animals in the MI group underwent baseline imaging 15 days before MI, had an MI induced on day 0 and were followed up during the acute stage at day 6 after MI and twice during follow-up i.e. at in week 5 at 34 days and in week 9 at 62 days post MI. The control group underwent CMR at the same time points as the MI group.

The MI group underwent one additional anaesthesia for induction of MI by 90 min occlusion of the LCX with a balloon catheter 15 days after baseline imaging (day 0). The additional anaesthesia and intervention were not performed in the control group.

### Imaging protocol

#### Functional imaging and strain analysis

Two- and four-chamber long-axis cine CMR was performed in addition to multi-slice short-axis cine imaging. Image acquisition was performed during ventilated breathing with the following imaging parameters: 1.8 × 1.8 mm^2^ spatial resolution, 8 mm slice thickness, 25 heart phases, 1.5 ms/3 ms TE/TR, 45° flip angle.

LV end-diastolic and end-systolic volumes (LVEDV/LVESV), ejection fraction (LVEF) as well as stroke volume (SV) were computed using commercial software (ISP, Philips Healthcare).

GRS, GCS and GLS were computed using Segment CMR (Medviso, Lund, Sweden).

#### Tagged imaging and evaluation of torsion

3D tagged imaging was performed acquiring three stacks [27] with orthogonal complementary spatial modulation of magnetization (CSPAMM) tagging [28] using the following imaging parameters: 2 × 2 × 5 mm^3^ spatial resolution, 110 × 110 × 110 mm^3^ field-of-view, 26 heart phases, 4 mm tagline distance, 4.3 ms/9.2 ms TE/TR, turbo factor 3, echo planar imaging (EPI) factor 7.

Tagged data was analysed using a 3D SinMod [29] based algorithm implemented in GTTagTrack (GyroTools LLC, Winterthur, Switzerland). For each slice position, a mid-mural contour was manually drawn and tracked over time. The rotation of each contour around the centre axis was plotted against the initial longitudinal position and a linear regression was consequently performed. The slope of the linear fit was computed to represent left-ventricular torsion/twist per unit length [30].

#### Mapping

T1 and T2 mapping were performed during suspended ventilation. T1 mapping was obtained using a modified Look-Locker inversion recovery sequence (MOLLI) [31] with the following imaging parameters: 2 × 2 mm^2^ spatial resolution, 8 mm slice thickness, 1.1 ms/2.3 ms TE/TR, 10–13 (pre-contrast) or 12–14 (post-contrast) inversion delays depending on the heart rate. T1 MOLLI was performed before and 20 min after administration of 0.2 mmol/kg gadolinium-based contrast agent. For T2 mapping a multi-echo spin-echo sequence with multi-shot EPI readout was acquired with the following parameters [32]: 2 × 2 mm^2^ spatial resolution, 10 mm slice thickness. Nine echoes with a ΔTE of 8.8 ms were sampled.

Native and post-contrast T1 maps, ECV maps as well as T2 maps were reconstructed upon image registration using an in-house Matlab (Mathworks, Natick Massachusetts, USA) tool [33] and manually segmented using GTVolume (GyroTools, Winterthur, Switzerland).

#### Cardiac diffusion tensor imaging

In-vivo cDTI was performed using a second-order motion-compensated spin echo sequence [34, 35]. The trigger delay (time between detection of R-wave and first radiofrequency pulse) was set to 65% of peak contraction [35–37] and data were acquired during ventilated breathing. Imaging parameters were: 2 × 2 mm^2^ spatial resolution, 8 mm slice thickness, TE/TR: 81 ms/5R–R intervals, three diffusion encoding directions @ b = 100 s/mm^2^, three diffusion encoding directions @ b = 200 s/mm^2^ and 12 diffusion encoding directions @ b = 450 s/mm^2^. Eight signal averages were obtained. In total 10 slices were acquired in packages of five slices in an interleaved fashion. The FOV was limited in phase encoding direction using non-coplanar excitation [38]. The total acquisition duration including preparation phases was approximately 28 min (at 60 bpm).

Diffusion parameters were computed using an in-house Matlab tool including non-rigid image registration and fitting the diffusion tensor to the cDWI data. MD, FA as well as the transmural change in helix angle (HA) were quantified. The HA was computed after projection of the tensor’s first eigenvector onto the longitudinal–circumferential surface as the angle between the resulting projection and the local circumferential direction according to [37]. The local radial, circumferential and longitudinal directions were calculated as described in [37]. Due to the thin wall at the location of MI, the transmural change of HA was computed in the remote area only i.e. in the septal segment (corresponding to American Heart Association segments 8 and 9 [39]). The myocardium was carefully segmented to avoid inclusion of right ventricular tissue [40].

#### Late gadolinium enhancement

LGE scar imaging was performed with a 3D inversion-recovery gradient-echo sequence approximately 15 min after contrast injection. The inversion time was adjusted to null non-infacted myocardial signal. Imaging parameters were: 2 × 2 × 8 mm^3^ spatial resolution, 1.4 ms/2.8 ms TE/TR, turbo factor 20.

Infarct mass was quantified using clinical software (ISP, Philips Healthcare) based on intensity thresholding at five standard deviations of non-enhanced remote tissue signal followed by manual refinement. Scar mass is reported as percentage of the total myocardial mass.

#### Statistics

Scalar values were averaged across the region of interest (infarction/remote zone) and statistically significant differences were tested for by either paired (infarction vs. remote zone in the same animal) or unpaired (remote zone in infarct animals vs. control animals) two-tailed Student’s t-test. A *p*-value smaller than 0.05 was considered statistically significant.

Diffusion (MD, FA) parameters were correlated to relaxometry (T1, T2) parameters and ECV values. Similarly, transmural change in HA was compared to strains and torsion.

For relaxometry mapping and diffusion parameters, the relative change in parameter was computed as paired difference between remote and infarcted area normalized by the values of the remote area. In order to evaluate any potential bias in the remote area, values were compared to healthy control data. To this end, differences between the remote area in infarcted animals and the same area in control animals were computed for all possible pairs of control and infarcted animals. The reported differences are normalized to the average of control and remote data.

## Results

All animals showed normal growth and weight gain during the longitudinal study programme: from 32 ± 2/29 ± 4 kg (baseline at − 15 days) to 43 ± 4/41 ± 6 kg (day 6), 58 ± 5/56 ± 5 kg (week 5) and 73 ± 6/72 ± 6 kg (week 9) for control/MI groups. LV mass, LVEF, LVEDV, LVESV and SV as well as hsTnT are listed in Table 1.

**Table 1:** Physiological parameters for control/infarct groups: left ventricular (LV) mass, left ventricular ejection fraction (LVEF), LV end diastolic volume (LVEDV), LV end systolic volume (LVESV), stroke volume (SV) and high sensitivity Troponin T

Control/infarct	Baseline	Day 6	Week 5	Week 9
LV mass (g)	51 ± 7/43 ± 2	58 ± 8/51 ± 9	71 ± 10/62 ± 11	85 ± 8/77 ± 12
LVEF (%)	51 ± 3/49 ± 4	54 ± 2/47 ± 3*	56 ± 1/43 ± 7*	54 ± 2/47 ± 2*
LVEDV (ml)	97 ± 13/85 ± 6	100 ± 12/108 ± 13	124 ± 17/122 ± 14	149 ± 5/152 ± 16
LVESV (ml)	48 ± 6/43 ± 4	49 ± 7/57 ± 9	54 ± 9/69 ± 6*	68 ± 4/81 ± 8*
SV (ml)	50 ± 7/42 ± 5	54 ± 5/51 ± 4	70 ± 9/53 ± 13	81 ± 4/71 ± 10
hs-Troponin T (ng/l)	8.0 ± 2.7/9.0 ± 5.4	9.8 ± 7.0/130.8 ± 62.6*	4.5 ± 1.7/7.2 ± 2.7	5.3 ± 2.3/14.0 ± 11.5

The asterisk indicates statistical significance (p < 0.05) comparing control and infarction

LV mass, LVEDV, LVESV as well as SV continuously increased over the course of the experiment in both study cohorts. While LVEF remained in a range of 51–56% in the control group, it was reduced to 43–47% after MI. The reduction in LVEF in the MI group was associated with continuously increasing LVESV over the duration of the experiment, above the values expected from growth in the controls. The relative size of the ischemic region based on LGE was 11 ± 4% (day 6), 12 ± 4% (week 5) and 11 ± 4% (week 9). The corresponding infarct mass was: 5.7 ± 2.5 g (day 6), 7.6 ± 3.0 g (week 5) and 8.8 ± 4.0 g (week 9).

**Fig. 1:**
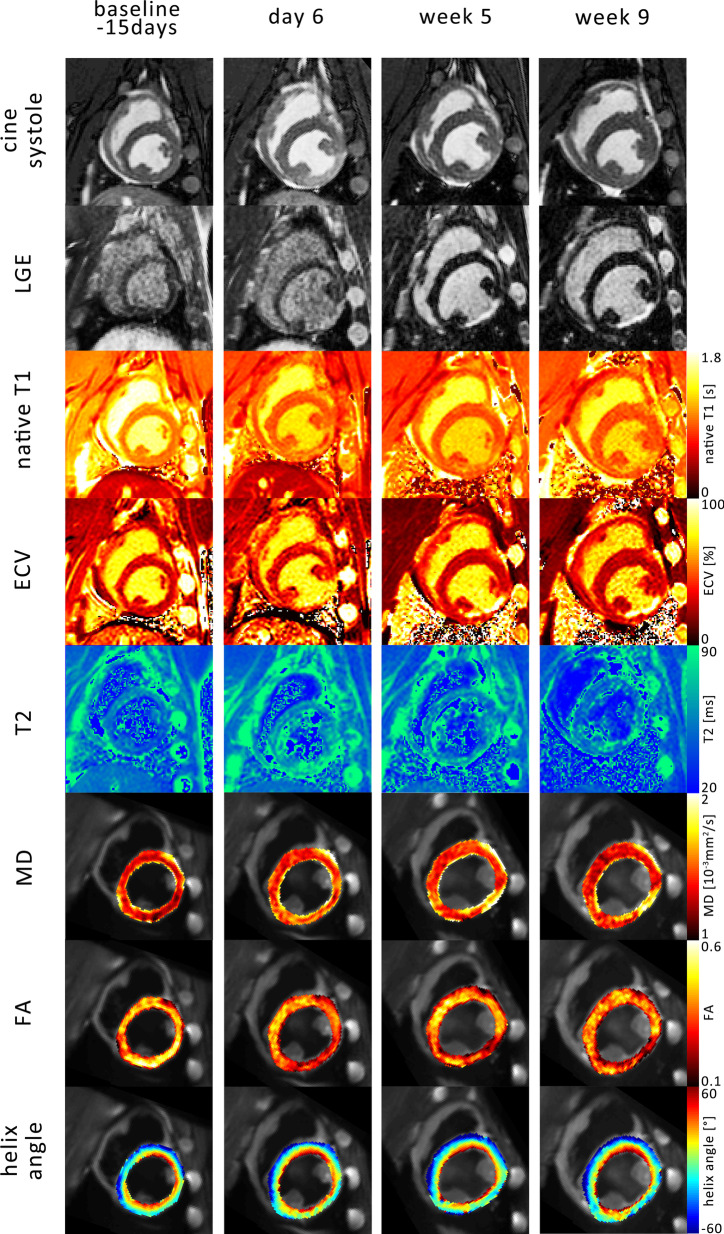
Example images over the time course of the experiment: baseline (15 days before infarction), 6 days, 5 weeks and 9 weeks after myocardial infarction. The systolic timeframe shows hypo-contraction in the inferior lateral wall coinciding with areas of late gadolinium enhancement (LGE) and changes in native T1, extra cellular volume fraction (ECV), T2, mean diffusivity (MD) and fractional anisotropy (FA)

Figure 1 shows example images of one infarct study animal over the time course of the experiment: baseline (15 days before MI) and 6 days, 5 weeks and 9 weeks after MI. The end-systolic time frame of the cine image series showed an area of hypo-contraction in the inferior lateral wall between the papillary muscles, which coincides with LGE signal enhancement. T2 values were elevated in the ischemic region at day 6 and correspondingly ECV maps showed clear enhancement during chronic follow-up. The areas of elevated MD and reduced FA were co-located with the infarcted segment. The transmural variation of myocyte aggregate orientation is depicted in the HA maps. While the remote area in the septum showed the characteristic continuous transmural change of HA, the far thinner area of MI did not present this trend as distinctly.

**Fig. 2:**
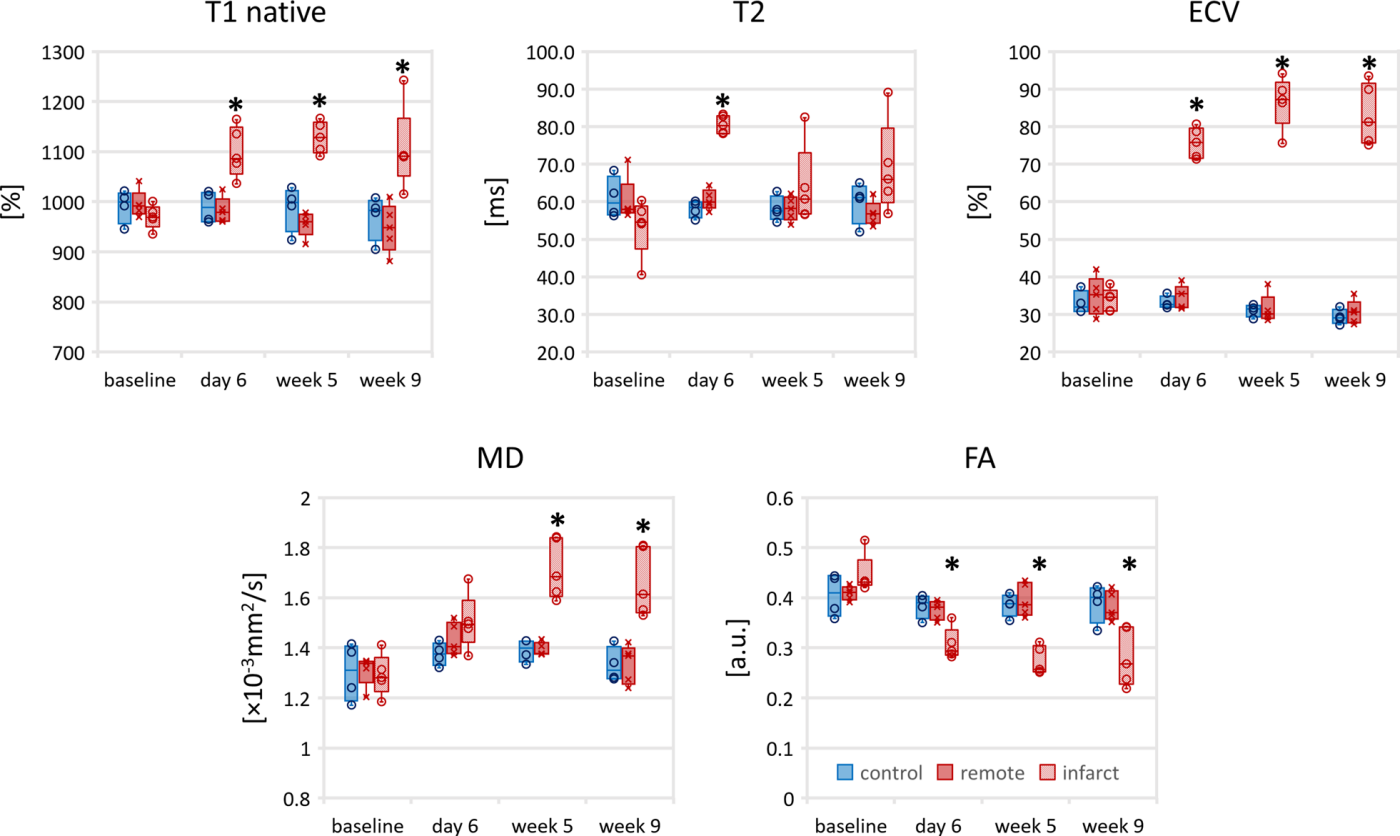
Progression of relaxivity (native T1, T2, ECV) as well as diffusion parameters (MD, FA) for the control group (blue), the remote area in the infarct group (red) and the infarct area (red shaded). The box corresponds to the center 50% and the error bars the minimum/maximum. The asterisk indicates statistical significance (p < 0.05) comparing to baseline (infarct zone)

Figure 2 shows the time evolution of native T1, ECV, T2, MD and FA. No significant differences were found between control group and the remote region in the MI group for native T1, ECV, T2, MD and FA. Native T1, ECV and MD were significantly elevated in the infarcted region and FA was significantly reduced (Additional file 1: Table S1) with respect to the remote region and control group. During the transition from acute to chronic MI stage, native T1 within the infarcted area remained in a range of 1099–1128 ms (change < 3% from acute to chronic). On the contrary, ECV as well as MD increased from the acute to the chronic stage by 14% (ECV), 14% (MD) and FA decreased by 11%. T2 as a marker for myocardial edema was significantly increased in the ischemic area only during the acute MI stage (81 ± 2 ms infarction vs 58 ± 2 ms control p < 0.001 and 61 ± 2 ms in the remote area p < 0.001) and decreased towards baseline values in all but one animal during chronic MI/follow-up studies.

**Fig. 3:**
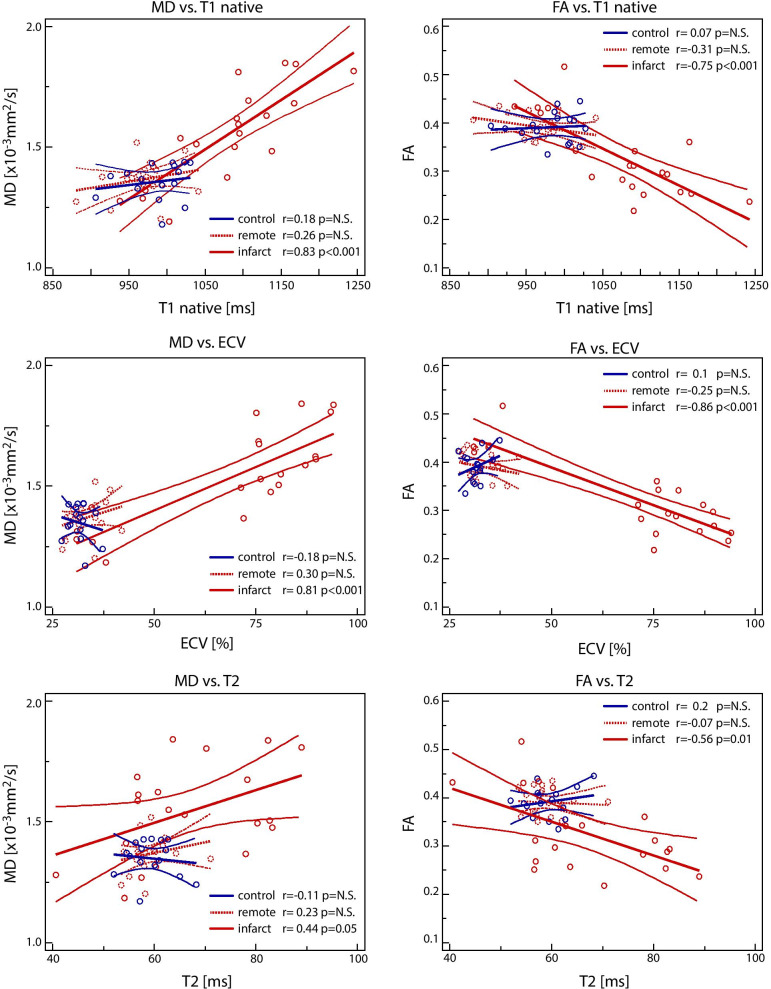
Correlation plots of relaxometry (native T1, ECV, T2) vs. diffusion parameters (MD, FA) over all time points. The blue lines indicate the control group, the red dotted lines the remote area in the infarct group and the red straight lines the infarcted area. Linear regression is plotted including the 95% confidence interval

The correlation results of parametric mapping and diffusion parameters for control/remote/infarct over all time points are presented in Fig. 3. MD correlated well with native T1 and ECV (r = 0.83 and 0.81 respectively with p < 0.001) and less with T2 (r = 0.44 with p = 0.05) in the infarcted region. FA was inversely correlated to native T1 (r = − 0.75 with p < 0.001), ECV (r = − 0.86 with p < 0.001) and T2 (r = − 0.56 with p < 0.01) in the infarcted zone. There was no correlation of parameters found in the control or remote region. Corresponding correlation analysis pooling all regions and time points as well as scatter plots for individual time points and regions are shown in Additional file 1: Figures S1–S3.

**Fig. 4:**
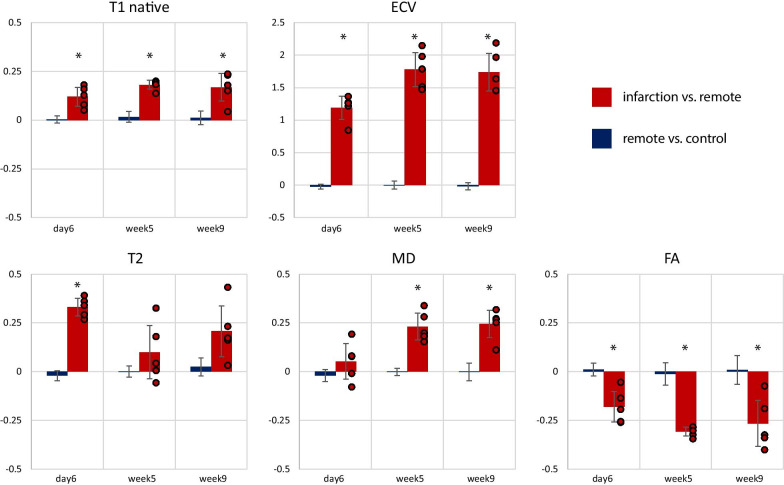
Relative change in native T1, extra cellular volume (ECV), T2, mean diffusivity (MD) and fractional anisotropy (FA) in the infarcted area compared to the remote area (red). The blue area depicts the difference between the remote area in infarcted hearts and healthy control data. Error bars indicate one standard deviation across cases. The asterisk indicates statistical significance (p < 0.05) comparing to baseline contrast

To compare the sensitivity of non-gadolinium-enhanced readouts to gadolinium-enhanced imaging, Fig. 4 shows the results of the relative parameter difference between infarcted and remote areas for native T1, ECV, T2, MD and FA. Correspondingly, the comparison of heathy control data and the remote area of infarcted hearts showed no bias. ECV showed the highest sensitivity with a 174–178% increase in the infarcted area relative to the remote zone during the chronic stage. Considering non-gadolinium enhanced data, T2 showed the largest relative change between infarct and remote during the acute MI stage (+ 33 ± 4% day 6, with p = 0.013 T2 vs. MD, p = 0.009 T2 vs. FA and p = 0.01 T2 vs. T1) while FA exhibited the largest relative change between infarct and remote during the chronic MI follow-up (+ 31 ± 2% week 5, with p = N.S. FA vs. MD, p = 0.03 FA vs. T2 and p = 0.003 FA vs. T1). Overall, MD as well as FA showed a higher contrast (> 23% for MD and > 27% for FA) during follow-up compared to native T1 (17–18%) and T2 (10–20%). The difference between FA and native T1 was statistically significant for both follow-up time points (p < 0.01) and for MD only for week 9 (p < 0.05). Similarly, the comparison of FA and T2 reached statistical significance for both follow-up time points (p < 0.01), while MD was only statistically significantly different for week 5 (p < 0.05). During the acute stage, diffusion and relaxometry parameters were all significantly different from other time points.

**Fig. 5:**
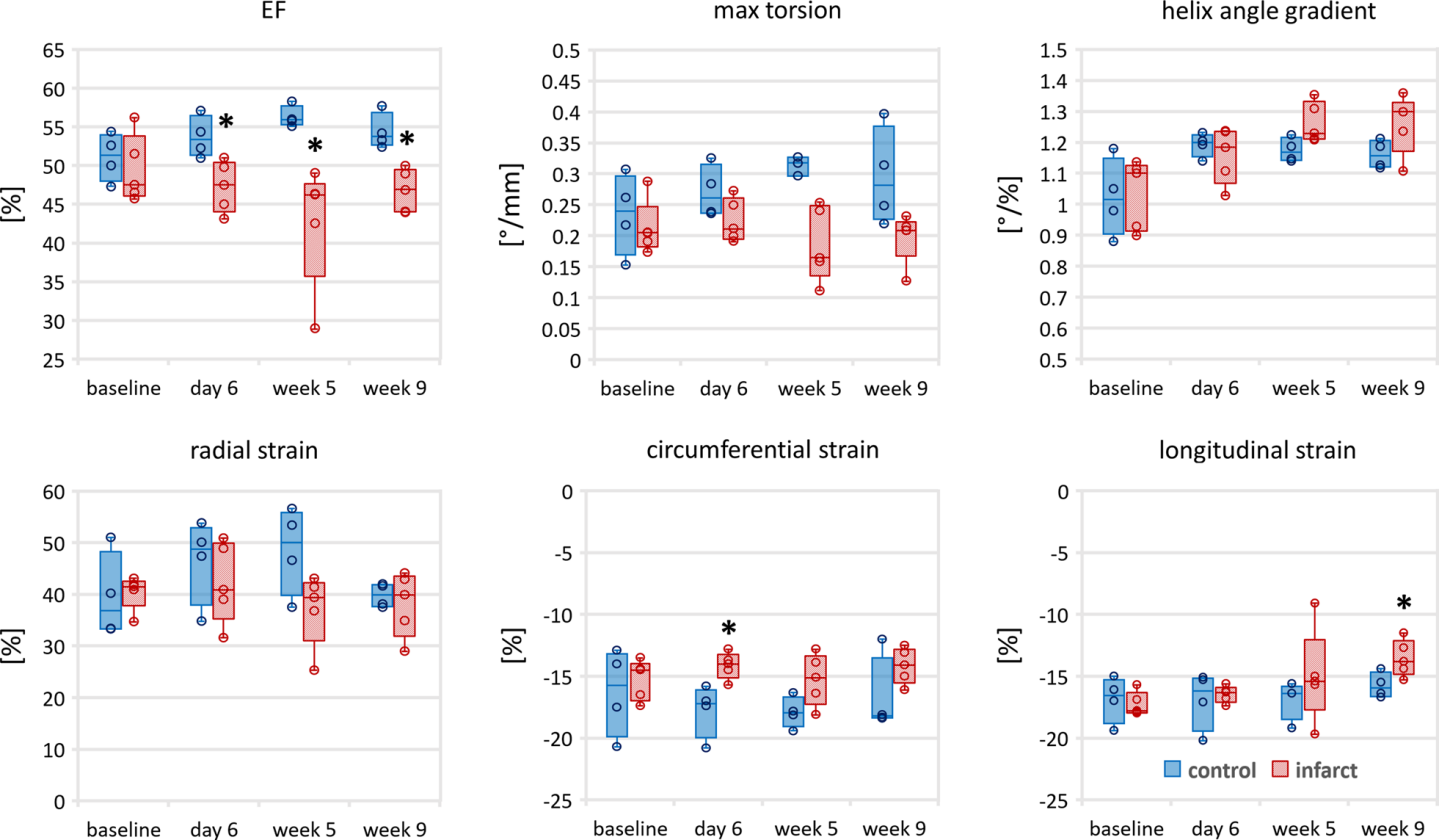
Temporal evolution of functional (ejection fraction (EF), maximum torsion, global radial/circumferential/longitudinal strain) as well as transmural helix angle gradient (remote zone/septum) for the control (blue) and infarct (red shaded) cohort. The box corresponds to the center 50% and the error bars the minimum/maximum. The asterisk indicates statistical significance (p < 0.05) comparing to baseline (infarct zone)

The analysis of change of functional and structural parameters such as LVEF, global myocardial strains, torsion and transmural HA gradient is shown in Fig. 5 and the corresponding values are reported in Table 2.

**Table 2:** Global radial, circumferential and longitudinal strains as well as torsion of the LV and transmural helix angle gradients of the remote (septal) region

	Global radial strain (%)	Global circumferential strain (%)	Global longitudinal strain (%)	Torsion (°/mm)	Helix angle gradient (°/%)
Control animal					
Baseline	39 ± 7	− 16 ± 3	− 17 ± 1	0.23 ± 0.05	− 1.02 ± 0.11
Day 6	47 ± 7	− 18 ± 2	− 17 ± 2	0.26 ± 0.03	− 1.19 ± 0.03
Week 5	49 ± 7	− 18 ± 1	− 17 ± 1	0.31 ± 0.03	− 1.17 ± 0.03
Week 9	40 ± 2	− 17 ± 3	− 16 ± 1	0.28 ± 0.05	− 1.16 ± 0.04
Infarct animal					
Baseline	40 ± 3	− 15 ± 1	− 17 ± 1	0.20 ± 0.03	− 1.04 ± 0.10
Day 6	42 ± 7	− 14 ± 1	− 16 ± 1	0.23 ± 0.05	− 1.16 ± 0.08
Week 5	37 ± 6	− 15 ± 2	− 15 ± 3	0.20 ± 0.06	− 1.26 ± 0.06
Week 9	38 ± 6	− 14 ± 1	− 14 ± 1	0.21 ± 0.02	− 1.26 ± 0.08

In the control cohort, LVEF, GRS and GCS increased in magnitude from baseline to week 5 (increase by 10% [2% to 18%] EF; 22% [5% to 69%] GRS; 10% [− 6% to 37%] GCS) followed by a decrease towards the final time point. This trend is not mirrored in the MI group as LVEF drops (− 14% [+ 3% to − 37%] week 5).

The transmural HA gradient remained constant from day 6 until the end in the control group, but continued to become steeper in the MI group until week 5 (9% increase from day 6 to week 5). From week 5 to 9 there was no change found in the MI group.

**Fig. 6:**
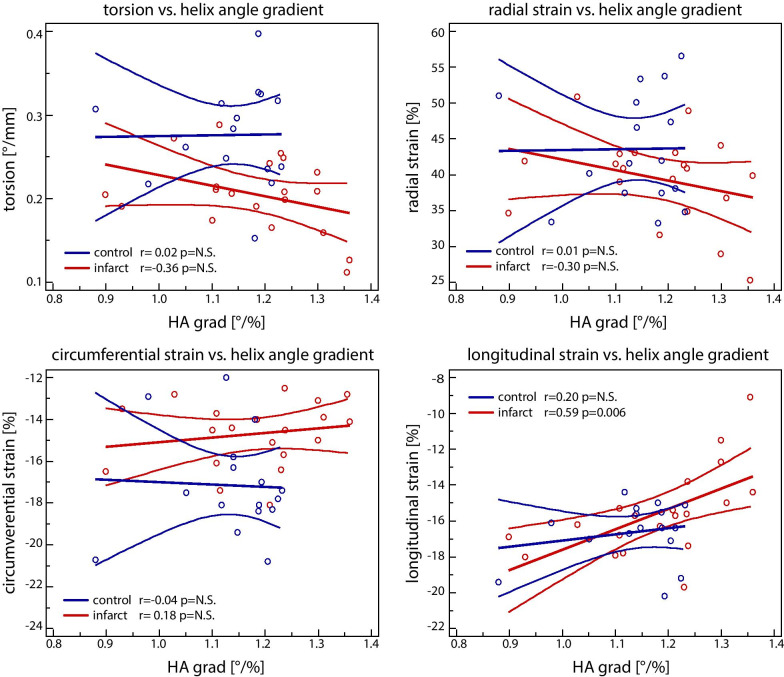
Correlation of torsion and strains with remote (septal) transmural helix angle gradient (HA grad) for the control (blue) and the infarcted (red) group

The corresponding correlation plots are shown in Fig. 6. While radial strain and torsion (Fig. 5) showed changes during disease progression, no significant correlation was found with the transmural HA gradient in the remote zone. However, a significant correlation between longitudinal strain and transmural HA gradient was detected (r = 0.59 with p < 0.05).

Correlation with relative scar mass showed a fair correlation with longitudinal strain (R = 0.48 p < 0.032) and good correlation with transmural HA variation (R = 0.71 p < 0.001).

## Discussion

In this study, we observed functional and structural changes after experimental MI. Edema during the acute MI stage was captured by elevated T2 values, while elevated native T1, ECV, MD and reduced FA values were found in the infarcted region during chronic MI follow-up. A reduction of ventricular torsion as well as longitudinal strain was present during the chronic stage together with a trend towards a steeper transmural HA distribution in the remote area.

Upon ischemia, a bimodal occurrence of edema has been reported in swine and humans [6, 41]. The deferred edema peaking at approximately 1 week post-MI has been attributed to healing processes accompanied by elevated T2 values in the ischemic region [42]. We found elevated T2 values during the acute stage (day 6) that converged back to baseline values during the chronic stage, in line with previous work [6]. The presence of edema during the acute stage coincided with an increase in MD and a decrease in FA. The increase in MD and reduction in FA in the ischemic region are attributed to the increase of ECV due to edema. Interestingly, FA and ECV exhibited a larger change in magnitude from baseline to acute stage, compared to the transition of acute to chronic stage, while the change in MD was larger in the chronic stage. Consequently, a higher correlation between ECV and FA was found compared to ECV and MD. The finding suggests that MD is more sensitive to chronic changes during scar formation, rather than to acute tissue swelling. During the acute MI stage, T2 mapping showed the largest difference between infarcted and remote regions emphasizing its value in identifying edema.

The chronic MI stage was reached by week 5 with absence of edema as T2 values normalized close to baseline values. ECV, MD and FA significantly increased in week 5 with a reduction of MD and ECV in week 9, as well as a significant decrease in FA corresponding to scar formation. Increased values of MD and decreased values of FA within scarred regions have previously been reported in in-vivo [43, 44] and ex-vivo [45] cDTI. While swelling of the interstitium and cardiomyocytes during the acute stage leads to a longer free path for water diffusion, full disintegration of cell membranes in the scarred region leads to fewer diffusion restrictions and structural disorder reflected in elevated MD and reduced FA values.

Interestingly, diffusional parameters showed a larger relative change during chronic follow-up compared to both non-contrast methods: native T1 and T2 mapping. The underlying change in tissue properties due to scar formation was identical for both parametric mapping and cDTI. However, imaging physics differ. While native T1 and T2 imaging probe changes in spin–lattice and spin–spin interaction, MD reflects changes in viscosity of the imaged liquid as well as the mean free pathway water molecules can diffuse without restriction as imposed by semipermeable and non-permeable membranes. Correspondingly, FA is predominantly probing changes in diffusion measurements as a function of spatial orientation and hence the overall anisotropy of tissue architecture. Our results suggest a significantly higher sensitivity to changes in myocardial microstructure by cDTI. However, if contrast agents were used, ECV presented the most prominent changes in contrast favouring contrast agent-based imaging over native-contrast diffusion imaging and relaxometry mapping.

In this study, the the LCX was occluded leading to an infarction area within the lateral wall of the LV. The septum was consequently chosen as remote zone.

Up to week 5, an increase of LVEF, torsion as well as radial strain was found in the control cohort that may be attributed to animal growth. In the control group, the transmural HA gradient only increased within the first 21 days (baseline to day 6), remained unchanged for the remainder of the experiment. In the MI group, this trend was not seen, as LVEF decreased immediately after induction of MI reflecting acute loss of function. The acute changes in function were followed by structural remodelling in the remote zone during chronic follow-up up to week 5. With structural remodelling, torsion, radial as well as longitudinal strain reduced in magnitude. The correlation analysis suggests a link between the structural changes in the remote zone and the loss of longitudinal strain. A reduction in LGS has been shown to correlate with poor outcome [46, 47] and LV remodelling [48].

Biomechanical modelling that was performed as part of a study on patients suffering from dilated cardiomyopathy revealed an increase in GLS associated with a steeper HA [49]. To this end, the trend towards elevated transmural HA variation may reflect a compensatory mechanism to address the lack of long-axis shortening in the presence of non-contractile infarcted tissue. During the early chronic follow up (week 5), GLS was comparable to the control cohort despite the presence of MI and an elevated HA was found. The increase in transmural HA, however discontinued after week 5 coinciding with a reduction of longitudinal strain potentially indicating onset of heart failure.

In a previous study on the comparison of LAD and LCX occlusion in swine with 1 and 3 months follow-up, a reduction in LVEF, GRS, GCS, GLS as well as torsion has been reported, when compared to a control group [50]. In line with our study, the authors did not find a correlation of radial/circumferential strain with clinical parameters such as LVEF, LVESV and scar size, while they found moderate correlation of these parameters with torsion and strong correlation with GLS.

### Limitations

While trends in changes of functional as well as structural parameters have been found, the study cohort is too small to conclude on statistical significance on some of the trends.

Control animals did not undergo a sham procedure.

Occlusion of the LCX was chosen as it was deemed to be tolerated better by the animals and lead to fewer dropouts. However, the affected area was located in the lateral wall between the papillary muscles. Both papillary muscles tend to stabilize the wall during contraction, potentially reducing the overall burden to the heart, when compared to large anterior wall infarctions. This may also be a reason why no significant changes in LV shape were observed.

A dependency of LV rotation [19], ventricular shape remodelling and dysfunction [51] on the location of MI has been reported. This indicates a potential dependency of myocardial response to the site of coronary occlusion and hence different microstructural remodelling.

While histology has been the standard method for validation of cDTI, more recently high resolution synchrotron imaging of entire rat hearts has been used to confirm the microstructural findings [52]. Neither methods have been available for this study. One key limiting factor compared to such ex-vivo measurements is the limited spatial resolution of in-vivo imaging.

Furthermore, CMR imaging had to be performed in different heart phases due to sequence requirements (e.g. DTI in systole and T2 mapping in diastole). The use of different imaging readouts (e.g. single shot EPI vs. multi shot gradient/spin echo) resulted in different off-resonance related image artefacts, causing geometrical and intensity distortions predominantly in the vicinity of the posterior vein [53]. This required manual segmentation of regions of interest according to anatomical landmarks and may add to additional variation.

## Conclusion

We longitudinally assessed the dynamics of MI from the acute to the chronic stage by functional and microstructural CMR imaging. Considering non-contrast enhanced methods, differentiation of infarcted and remote tissue based on diffusion parameters was superior when compared to native T1 and T2 mapping in the chronic stage, indicating a higher sensitivity of cDTI compared to relaxometry in response to microstructural alterations. Moreover, the correlation between microstructural changes and reduced global contraction and rotation illustrates the multi-scale response of the heart to myofiber injury and scar formation. Therefore, the combination of functional and microstructural diffusion-based CMR imaging is considered a valuable tool to characterize MI across spatial scales.

## Supplementary Information

**Additional file 1: Table S1.** Native T1, T2, extra cellular volume (ECV), mean diffusivity (MD) and fractional anisotropy (FA) for healthy controls and for the remote as well as infarcted zone of the infarct cohort. The asterisk indicates statistically significant differences between infarcted and remote regions (p < 0.05). **Fig. S1.** Pearson correlation of all time points and all regions (control/remote/infarction). The asterisk indicates a p-value < 0.001. ECV, extracellular volume fraction; MD, mean diffusivity, FA, fractional anisotropy. **Fig. S2.** Scatter plots plotting mean diffusivity (MD) against extra cellular volume (ECV), T1 native and T2 for each region (control/remote/infarct) and each time point individually. **Fig. S3.** Scatter plots of fractional anisotropy (FA) against extra cellular volume (ECV), native T1 and T2 for each region (control/remote/infarct) and for each time point individually.

## Data Availability

Upon reasonable request to the corresponding author.

## Abbreviations

BW: Body weight
cDTI: Cardiac diffusion tensor imaging
CMR: Cardiovascular magnetic resonance
CSPAMM: Complementary spatial modulation of magnetization
ECG: Electrocardiogram
ECV: Extracellular volume fraction
EPI: Echo planar imaging
FA: Fractional anisotropy
GCS: Global circumferential strain
GLS: Global longitudinal strain
GRS: Global radial strain
HA: Helical angle
hsTNT: High sensitivity troponin T
LAD: Left anterior descending coronary artery
LCX: Left circumflex coronary artery
LGE: Late gadolinium enhancement
LV: Left ventricle/left ventricular
LVEDV: Left ventricular end-diastolic volume
LVEF: Left ventricular ejection fraction
LVESV: Left ventricular end-systolic volume
MACE: Major adverse cardiac events
MD: Mean diffusivity
MI: Myocardial infarction
MOLLI: Modified Look-Locker inversion recovery
SV: Stroke volume
TE: Echo time
TR: Repetition time
