# Supplementary material for: Cardiovascular magnetic resonance imaging of functional and microstructural changes of the heart in a longitudinal pig model of acute to chronic myocardial infarction
Source: J Cardiovasc Magn Reson. 2021 Sep 20;23:103. doi: 10.1186/s12968-021-00794-5 (PMC8451129; doi:10.1186/s12968-021-00794-5)
Supplement: Supplementary file 1 — Additional file 1: Table S1. Native T1, T2, extra cellular volume (ECV), mean diffusivity (MD) and fractional anisotropy (FA) for healthy controls and for the remote as well as infarcted zone of the infarct cohort. The asterisk indicates statistically significant differences between infarcted and remote regions (p < 0.05). Fig. S1. Pearson correlation of all time points and all regions (control/remote/infarction). The asterisk indicates a p-value < 0.001. ECV, extracellular volume fraction; MD, mean diffusivity, FA, fractional anisotropy. Fig. S2. Scatter plots plotting mean diffusivity (MD) against extra cellular volume (ECV), T1 native and T2 for each region (control/remote/infarct) and each time point individually. Fig. S3. Scatter plots of fractional anisotropy (FA) against extra cellular volume (ECV), native T1 and T2 for each region (control/remote/infarct) and for each time point individually. [file 12968_2021_794_MOESM1_ESM.docx]

|  |  | native T1 [ms] | T2 [ms] | ECV [%] | MD  [×10^-3^mm^2^/s] | FA |
| --- | --- | --- | --- | --- | --- | --- |
| control | baseline | 991±29 | 61±5 | 33±3 | 1.30±0.10 | 0.41±0.04 |
|  | day 6 | 989±28 | 58±2 | 33±1 | 1.37±0.04 | 0.38±0.02 |
|  | week 5 | 987±39 | 58±3 | 31±1 | 1.39±0.04 | 0.39±0.02 |
|  | week 9 | 969±39 | 59±5 | 29±2 | 1.33±0.06 | 0.39±0.03 |
| remote zone | baseline | 995±24 | 60±5 | 35±5 | 1.31±0.05 | 0.41±0.01 |
|  | day 6 | 982±23 | 61±2 | 35±3 | 1.43±0.06 | 0.38±0.02 |
|  | week 5 | 956±22 | 58±3 | 31±3 | 1.39±0.02 | 0.40±0.03 |
|  | week 9 | 947±43 | 57±3 | 31±3 | 1.34±0.07 | 0.38±0.03 |
| infarct zone | baseline | 970±21 | 53±7 | 34±3 | 1.29±0.07 | 0.45±0.04 |
|  | day 6 | 1099±45* | 80±2* | 76±4* | 1.50±0.10 | 0.31±0.03* |
|  | week 5 | 1128±28* | 64±10 | 87±6* | 1.72±0.11* | 0.27±0.03* |
|  | week 9 | 1106±74* | 69±11 | 83±7* | 1.66±0.12* | 0.28±0.05* |

Table S1: native T1, T2, extra cellular volume (ECV), mean diffusivity (MD) and fractional anisotropy (FA) for healthy controls and for the remote as well as infarcted zone of the infarct cohort. The asterisk indicates statistically significant differences between infarcted and remote regions (p<0.05).


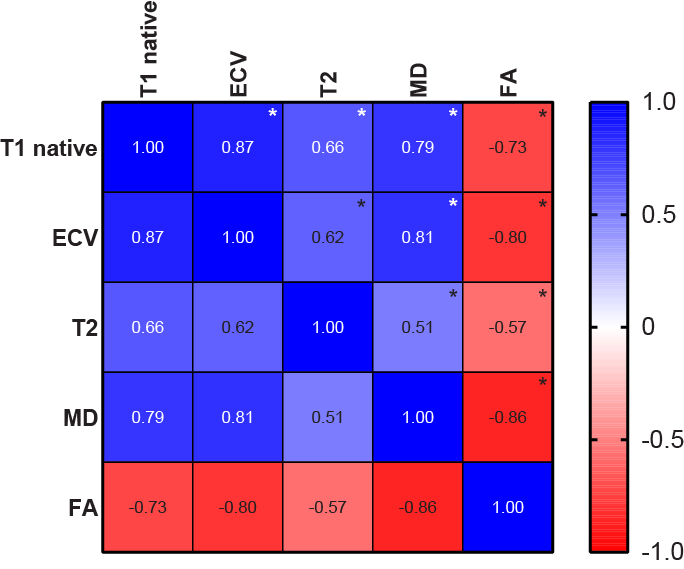


Fig S1. Pearson correlation of all time points and all regions (control/remote/infarction). The asterisk indicates a p-value < 0.001. ECV, extracellular volume fraction; MD, mean diffusivity, FA, fractional anisotropy


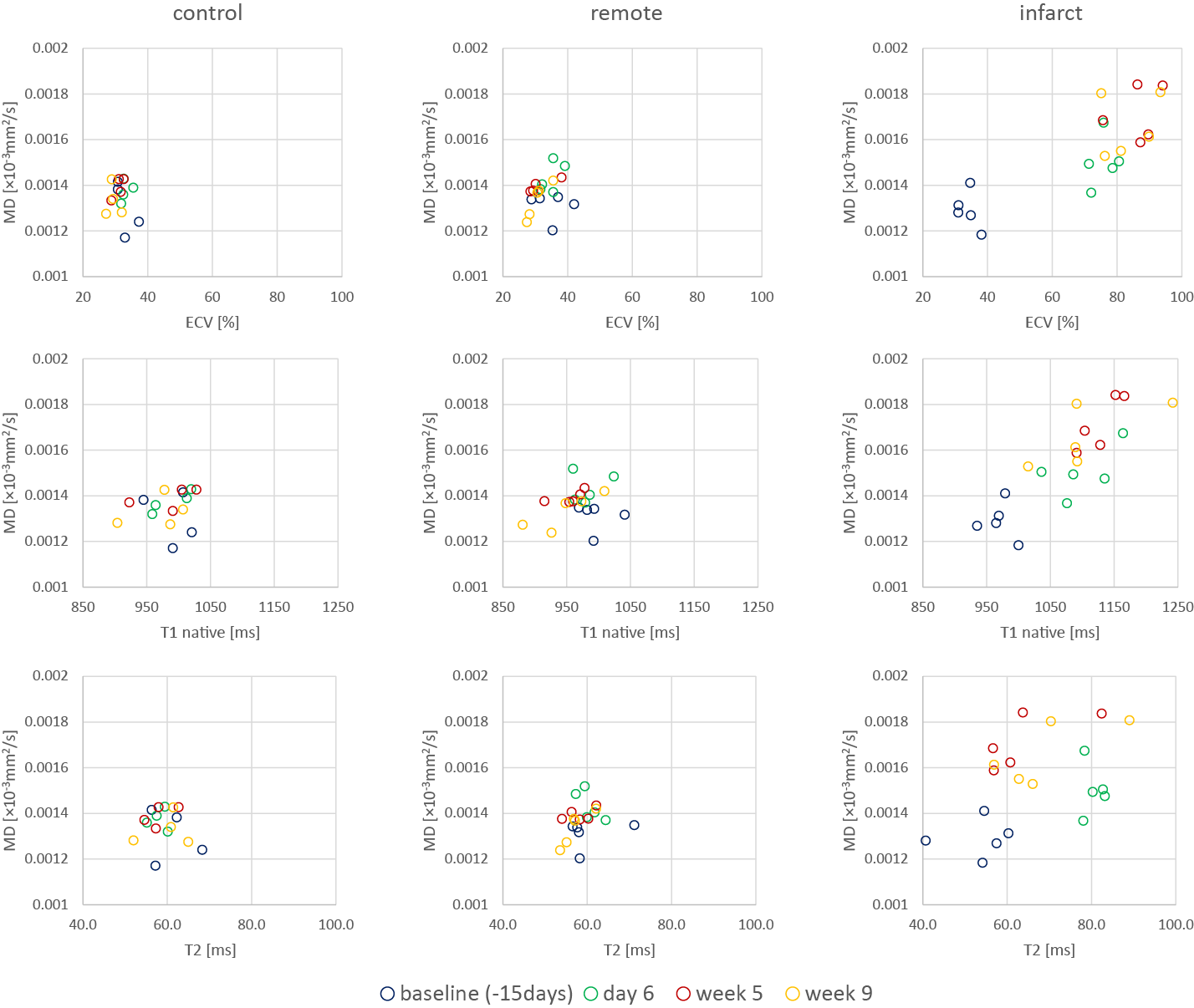


Fig S2. Scatter plots plotting mean diffusivity (MD) against extra cellular volume (ECV), T1 native and T2 for each region (control/remote/infarct) and each time point individually.


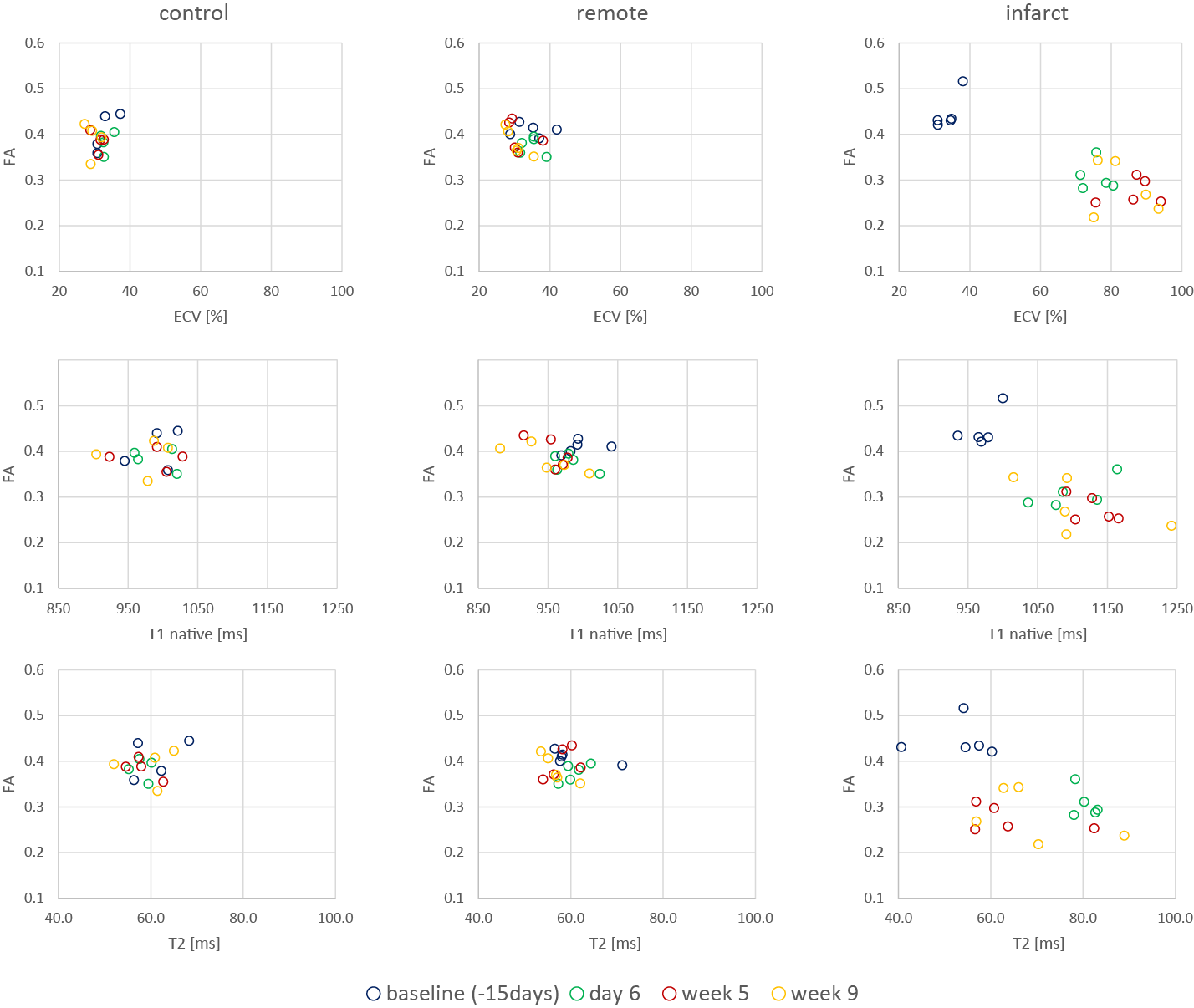


Fig S3. Scatter plots of fractional anisotropy (FA) against extra cellular volume (ECV), native T1 and T2 for each region (control/remote/infarct) and for each time point individually.
